# Novel SNP markers in *InvGE* and *SssI* genes are associated with natural variation of sugar contents and frying color in *Solanum tuberosum* Group Phureja

**DOI:** 10.1186/s12863-017-0489-3

**Published:** 2017-03-09

**Authors:** Diana Duarte-Delgado, Deissy Juyó, Christiane Gebhardt, Felipe Sarmiento, Teresa Mosquera-Vásquez

**Affiliations:** 1 Faculty of Agricultural Sciences, Agronomy Department, National University of Colombia, Bogotá, Colombia; 2 Present address: INRES-Plant Breeding, University of Bonn, Bonn, Germany; 3 Department of Plant Breeding and Genetics, Max Planck Institute for Plant Breeding Research, Cologne, Germany; 4 Faculty of Sciences, Biology Department, National University of Colombia, Bogotá, Colombia

**Keywords:** Association mapping, Potato frying quality, Carbohydrate metabolism, Candidate genes

## Abstract

**Background:**

Potato frying color is an agronomic trait influenced by the sugar content of tubers. The candidate gene approach was employed to elucidate the molecular basis of this trait in *Solanum tuberosum* Group Phureja, which is mainly diploid and represents an important genetic resource for potato breeding. The objective of this research was to identify novel genetic variants related with frying quality in loci with key functions in carbohydrate metabolism, with the purpose of discovering genetic variability useful in breeding programs. Therefore, an association analysis was implemented with 109 SNP markers identified in ten candidate genes.

**Results:**

The analyses revealed four associations in the locus *InvGE* coding for an apoplastic invertase and one association in the locus *SssI* coding for a soluble starch synthase. The SNPs *SssI*-*C*_*45711901*_ *T* and *InvGE*-*C*_*2475454*_ *T* were associated with sucrose content and frying color, respectively, and were not found previously in tetraploid genotypes. The rare haplotype *InvGE*-*A*_*2475187*_ *C*_*2475295*_ *A*_*2475344*_ was associated with higher fructose contents. Our study allowed a more detailed analysis of the sequence variation of exon 3 from *InvGE*, which was not possible in previous studies because of the high frequency of insertion-deletion polymorphisms in tetraploid potatoes.

**Conclusion:**

The association mapping strategy using a candidate gene approach in Group Phureja allowed the identification of novel SNP markers in *InvGE* and *SssI* associated with frying color and the tuber sugar content measured by High Performance Liquid Chromatography (HPLC). These novel associations might be useful in potato breeding programs for improving quality traits and to increase crop genetic variability. The results suggest that some genes involved in the natural variation of tuber sugar content and frying color are conserved in both Phureja and tetraploid germplasm. Nevertheless, the associated variants in both types of germplasm were present in different regions of these genes. This study contributes to the understanding of the genetic architecture of tuber sugar contents and frying color at harvest in Group Phureja.

**Electronic supplementary material:**

The online version of this article (doi:10.1186/s12863-017-0489-3) contains supplementary material, which is available to authorized users.

## Background

Potato frying color is influenced by the sugar content of the tubers, because the hydrolysis of sucrose by invertase is the main source of the reducing sugars glucose and fructose, which are precursors of the Maillard reaction. This reaction produces dark pigments and toxic products such as acrylamide during the non-enzymatic reaction between reducing sugars and free amino acids at high temperatures [1, 2]. The high content of reducing sugars in tubers causes a low quality of fried potato products. The biotic and abiotic stresses present during plant development, the temperatures during the vegetative growth, and the genotypic differences in carbohydrate metabolism are crucial factors that influence the sugar contents of tubers at harvest [3, 4]. Sucrose, glucose and fructose are important metabolic signals in transduction pathways that affect the expression of several classes of genes involved in all stages of plant development and related to stress responses. Therefore, the sugar content of storage organs such as potato tubers is a complex trait controlled by multiple genetic and environmental factors [5–8]. Consequently, it is of particular interest to identify variants of genes related to low tuber sugar content at harvest. Such variants might be useful diagnostic markers in breeding programs aimed at improving frying quality.

Association mapping is a strategy to study the genetic basis of complex traits that has advantages compared to linkage mapping for the discovery of diagnostic molecular markers [9]. This strategy allows the mapping of quantitative traits more precisely due to the use of natural populations or diversity panels, where there are higher numbers of ancient recombination events. Consequently, linkage disequilibrium (LD) decays faster than in a bi-parental segregating population. Therefore, the mapping resolution is increased because of the reorganization of the chromosomes in smaller regions [10, 11]. Association mapping is also advantageous because it analyses the effects of multiple alleles in a single experiment. This is important for the efficient development of diagnostic molecular markers for traits of interest in breeding populations, with the purpose of contributing to the selection of genotypes with the desired qualities [12].

There is a large body of knowledge on the metabolic pathways and enzymes involved in starch synthesis, degradation and transport in potato. Sixty nine functional genes in carbohydrate metabolism have been mapped to linkage maps of the 12 potato chromosomes [13]. Several of these candidate genes have been found to be associated with natural variation of frying color or reducing sugar content [7, 14–17]. These studies have revealed the multi-loci genetic architecture of sugar content and frying color related to genes coding for functional enzymes in carbohydrate metabolism in tetraploid breeding populations of potato (2*n* = 4 × = 48). To assess the diagnostic power of genes with key functions in carbohydrate metabolism in diverse germplasm, it is necessary to search for genetic variants associated with frying quality in different potato populations, with the purpose of discovering genetic variability useful in potato breeding.

*Solanum tuberosum* Group Phureja consists mainly of diploid genotypes (2*n* = 2 × = 24) with short day adaptation and tuber sprouting at harvest [18]. In Andean countries, Group Phureja constitutes an important crop, particularly for small farmers [19]. Genotypes with round tubers, yellow flesh and skin represent an interesting potential for widespread use due to their desirable organoleptic properties, nutritional quality, and processing potential [20–23]. This cultivated potato is also a valuable genetic resource for the introgression of agronomic traits into tetraploid potatoes from S. *tuberosum* subsp. *tuberosum* that are the most extensively grown w﻿orldwide [24–27]. The doubled-monoploid genotype DM1-3516 R44 from Group Phureja represents the potato reference genome [28]. This genome sequence has contributed to the understanding of the genetic basis of various traits in tetraploid potato [7, 29]. This genomic tool is also important for studying agronomic traits of Group Phureja such as frying quality.

The phenotypic assessment of potato frying quality has been performed mostly by means of visual scales of frying color in linkage and association mapping studies [7, 15, 30]. However, it is desirable to improve the phenotypic evaluations in order to obtain precise quantitative data that contribute to the finding of reliable variants associated with quantitative traits of interest [31, 32]. This can be achieved through the use of chromatographic methods which quantify the precursors of the Maillard reaction. HPLC is capable of rapid, specific, sensitive, and precise measurements of sugar contents in potato tubers [33, 34] and should be implemented in mapping studies.

Accessions from the Colombian Central Collection of Group Phureja (CCC) that constitute the Working Collection of the Breeding Program at the National University of Colombia have been characterized for their frying quality at harvest. Plants were cultivated in plots in highlands over 2800 meters above the sea level, where low temperatures favored the accumulation of reducing sugars undesirable for frying purposes [35]. Previously, frying color was assessed in tubers from experimental fields in nine environments using a visual scale. From this multi-environmental data, a chip darkening index was estimated for each genotype using a Bayesian approach [35]. Recently, a HPLC method was applied to measure sucrose, glucose, and fructose contents after harvest in the same accessions in a single-environment trial. Reducing sugars and chip darkening index showed moderate significant correlations which supports a positive relationship between the two measurements and that both data sets are consistent [36].

The purpose of the research presented here was to find allelic variants associated with sugar contents and frying color in a Group Phureja population using a candidate gene approach. Therefore, the phenotypic assessments aforementioned were used to implement an association mapping strategy in order to elucidate the molecular basis of frying quality in Phureja tubers. The association analysis was performed with SNP markers identified in candidate genes with key function in carbohydrate metabolism, which had shown associations with potato frying color and sugar content in tetraploid potatoes [7, 14–17, 37].

## Methods

### Plant material and phenotypes

A set of 108 diploid landrace accessions from the Working Collection of the Breeding Program at the National University of Colombia and four commercial diploid cultivars (Criolla Colombia, Criolla Latina, Criolla Galeras, and Criolla Guaneña) were used in this study. Chip color data collected in nine environments allowed the estimation of a multi-environmental chip darkening index in a previous study using a Bayesian approach [35]. The index was calculated through a scale that establishes equivalences between the International Potato Center color scale [38] and a chip darkening percentage [35]. An explanatory table of this scale with images is shown in [36]. The first degree with the lightest color represented a darkening percentage of 0, while the fifth degree with the darkest color represented a darkening percentage from 90 to 100. Tubers of the 112 genotypes were also assessed at harvest in a single environment for sucrose, glucose, fructose, reducing and total sugar contents, as well as for their glucose/fructose and sucrose/reducing sugars ratios [36]. Mean values of these eight variables (seven sugar measurements and chip darkening index) from each genotype were used for the independent association analysis of each trait.

### Candidate gene sequencing and SNP calling

Genomic DNA was isolated with DNeasy Plant Mini Kit™ (Qiagen) from young leaf tissue. DNA was quantified with a NanoDrop 2000c spectrophotometer (Thermo Scientific) and adjusted to a concentration of 10 ng μL^−1^ for the Polymerase Chain Reaction (PCR) amplification. A group of ten candidate genes previously reported with association to the frying quality trait in tetraploid populations were selected for this study: *InvGE* [15, 17, 39], *Pain*-*1* [15, 16, 39, 40], *Stp23* [7, 15, 16], *StpL* [7, 15, 16], *AGPaseS*-*a* [7, 15], *SssI* [7, 16], *GWD* [7, 41], *UGPase* [42], *Sps* [16], and *G6pdh* [16]. Three primer pairs were designed per gene using the potato reference genome (version 4.03) [28, 43] in the SPUD database [44] (http://solanaceae.plantbiology.msu.edu/cgi-bin/gbrowse/potato/). In addition, seven primer pairs were tested in the Group Phureja accessions in candidate gene regions analyzed by Fischer et al. [37] (*LapN* and *KT*-*InvInh*) and Schreiber et al. [7] (*BMY*-*8*/*2*, *PWD*, *AGPaseS*, *INV*-*8*/*2*, and *PGM*-*3*).

The primers in 16 genes were tested in eight random genotypes (Criolla Latina, CCC 21, CCC 52, CCC 59, CCC 116, CCC 123, CCC 127, and CCC 132) and those with appropriate quality of the amplicon sequence and with at least four SNPs were selected for amplifying and sequencing in the whole population (Table 1). PCR conditions were the same as described by Schreiber et al. [7]. Annealing temperatures of primers used in the population are specified in Table 1.

**Table 1:** Loci (PGSC0003DMG*********) from the reference genome [28, 43] analyzed in 112 *Solanum tuberosum* Group Phureja accessions

Gene Acronym	Locus	Chr.	Primer Sequences 5′–3′	Ta (°C)	Amplicon	No. SNPs Scored
(GenBank Accesion No.)					size (bp)	(Scored previously)^e^
*Stp23* (PHO1)^a^ (D00520)	PGSC0003DMG400007782	III	*f-cagatatgttacatactctacc	59	963	4 (0)
			r-tcattagtcacaactttatcgg			
*StpL* (PHO1)^a^ (X73684)	PGSC0003DMG400028382	V	*f-ttacattgcacaagcacaagc	57	982	14 (1) [67]
			r-gtgtacatacaatactctatcc			
*SssI* (Y10416)	PGSC0003DMG402018552	III	f-aacaataggaatttaccataacc	57	970	12 (0)
			*r-atattcccaaacaaaacagagc			
*InvGE* (INV-cw)^a^ (AJ133765)	PGSC0003DMG400008943	IX	f-caattcttcgattcttcatagg	57	798	9 (8) [39]
			*r-aattgaagcagatcatgtagg			
*Pain1* (INV)^a^ (X70368)	PGSC0003DMG400013856	III	f-catacattacactatagatcc	56	926	5 (2) [39, 40]
			*r-aattgaagcagatcatgtagg			
			f-caaaatgaatacatattaagagg^b^	56	707	4 (0)
			*r-cttaagcagttgcttagagc			
*UGPase* (D00667)	PGSC0003DMG401013333	XI	f-atgatgttctccacttaaaagc	56	805	11 (1) [42]
			*r-tttcagattttcagaagagagg			
			*f-tgattaacgatactatacgtcc	56	931	12 (1) [42]
			r-ttaaaacttccttatactatatgg			
*GWD* (Y09533)	PGSC0003DMG400007677	V	f-ttctgttatctacttagttacg	56	992	5 (3) [7, 41]
			*r-gttttatatcttgcttctttgg			
*BMY*-*8*/*2* (AF393847)	PGSC0003DMG400001855	VIII	f-gctactggacatggtgacaga^c^	57	560	9 (9) [5]
			*r-ttacatagaggtctgtcctgcttgag			
*PWD* (AY747068)	PGSC0003DMG400016613	IX	*f-ggtctgatgatctatctgattgc^c^	57	871	16 (16) [7, 41]
			r-gacatcttgaggagaaccaaactt			
*LapN* (X77015)	PGSC0003DMG400007831	XII	f-gcttcctggtcttggctc^d^	60	985	8 (3) [37]
			*r-gataggcatacgcagccaggtcagaaatcaa			

*Chr* chromosome, *Ta* annealing temperature

*Primer used for amplicon sequencing

^a^These parenthesis include the alternative acronyms used in the starch-sugar interconversion pathway scheme adapted by Schreiber et al. [7]

^b^From a region downstream of the *Pain1* candidate gene

^c^From Schreiber et al. [7]

^d^From Fischer et al. [37]

^e^The studies where the SNPs or the related amino acid changes were previously reported in tetraploid potatoes are indicated

Amplicons were visualized in 1.5% (w/v) agarose gels in 1× TAE (Tris-acetate-EDTA) buffer and stained with ethidium bromide (0.4 μg mL^−1^). PCR products were cleaned using ExoSAP-IT® (USB) and custom sequenced at the Max-Planck-Genome-Center Cologne using the dideoxy chain-termination sequencing method, an ABI PRISM Dye Terminator Cycle Sequencing Ready Reaction Kit, and an ABI PRISM 3730 automated DNA Sequencer (Applied Biosystems). SNPs were detected by visual examination of the sequence trace files for overlapping base calling peaks using Geneious Software version 8.0 (Biomatters). The SNPs were scored using Data Acquisition and Analysis Software (DAx) (Van Mierlo Software Consultancy); in the case of heterozygous individuals, overlapping base calling peaks were identified.

### Association analysis

Association analysis was carried out considering that the Group Phureja accessions studied do not have a marked population structure and present a fast LD decay (*r* ^*2*^ value of 0.1 in a distance range of 1 to 2.5 Mbp in individual chromosomes) [27, 45]. Markers included in the analysis were those with a minor allele frequency higher than 0.01. A compressed mixed linear model (model 1) [46] and an enriched compressed mixed linear model (model 2) [47] were implemented with GAPIT R Package (version 1) [48] for the analyses. These models accounted for population structure and an additional parameter of group kinship that allows the reduction of the random effect of kinship among genotypes and improves the statistical power [46, 47]. Quantile-quantile (QQ) plots of the expected and observed *F*-test probabilities for the SNP markers were assessed to identify the appropriate model for controlling type I errors caused by population structure and familial relatedness [49]. Accordingly, we choose from the two models the one with better adjustment for each phenotypic variable. To improve the adjustment of the association models of glucose and fructose contents, we removed the three genotypes with extreme values which constituted a single group as described by Duarte-Delgado et al. [36]. The QQ plots from the selected models of each variable with significant associations are shown in the Additional file 1.

The probability *p* for marker-trait associations was expressed on a -log_10_ scale. SNP markers with a *p*-value − log_10_ > 2 were considered significantly associated with the variable studied. To support the reliability of the associations found with the mixed model approach, an analysis of variance (ANOVA) was performed using a linear model implemented in Genstat software version 17 (VSN International). Differences between phenotypic means of the genotypic classes were detected considering a level of significance of α = 0.05. Therefore, markers with significant *p*-values showing effect in the phenotypic variables were retained and presented as associated. LD was estimated between pairs of SNP markers that were found with significant associations in the same chromosome, using the r^2^ statistic [50]. Annotated potato gene models from the SPUD database allowed the location of associated markers in intronic or exonic regions [44]. Amino acid changes caused by SNPs in exonic regions were predicted using the Open Reading Frame (ORF) Finder tool from NCBI [51].

## Results

The test of primers in 16 candidate genes allowed the sequence analysis of 12 amplicon sequences from ten genes in 108 Group Phureja accessions. This analysis led to the identification of 109 SNP markers, 44 of which were previously reported for tetraploid genotypes (Table 1). The position of these genes in the starch-sugar interconversion pathway can be observed in the scheme from Schreiber et al. [7]. Some enzyme acronyms that differ from those in the scheme are distinguished in the Table 1. The gen *LapN* is the exception, it is not shown in the pathway because it corresponds to a leucine aminopeptidase that was identified associated to the frying quality trait through a comparative proteomics approach [37]. From these candidate genes studied, five SNPs in two genes showed seven significant associations according to the mixed model and ANOVA analyses (Table 2). The positions of these SNPs are shown in the Additional file 2 in *InvGE* and *SssI* amplified sequences. The positions of the SNPs scored in the remaining eight genes studied are shown in the Additional file 3. Both additional files with the sequences indicate which markers correspond to the previously reported markers for tetraploid potatoes.

**Table 2:** SNP markers associated with sugar contents and chip darkening index in *Solanum tuberosum* Group Phureja

Gene-SNP^a^	Trait	Minor Allele	−log_10_(*p*-value)^b^	Percent variance
		Frequency, %		explained and effect^c^
*InvGE*-*C*_*2475454*_ *T*	Chip darkening index	2.0 (T)	2.4**	15.6 ↑
***InvGE***-***G***_***2475344***_ ***A*** ^*d*^	Fructose	9.4 (A)	2.2**	7.2 ↓
***InvGE***-***C***_***2475295***_ ***A***	Fructose	9.0 (C)	2.2***	7.0 ↓
***InvGE***-***G***_***2475187***_ ***A***	Fructose	9.0 (A)	2.7***	8.5 ↓
*InvGE*-*G*_*2475187*_ *A*	Glucose	9.0 (A)	2.0*	6.6 ↓
*InvGE*-*G*_*2475187*_ *A*	Total sugars	9.0 (A)	2.0***	8.8 ↓
*SssI*-*C*_*45711901*_ *T*	Sucrose	35.5 (C)	2.0*	13.5 ↑

^a^The SNP reference allele is followed by its position in the chromosome and the nucleotide from the allelic variant

^b^SNP markers with a − log_10_ (*p*-value) higher than two were considered with significant effect on the variables studied with the mixed model approach. The markers were confirmed with an ANOVA considering a level of significance of α = 0.05. *p*-values: ***, *p* ≤ 0.001; **, *p* ≤ 0.01; *, *p* ≤ 0.05

^c^The percent variance explained expresses the effect of the minor allele in the trait. Arrows indicate the direction of the effect on the trait, upwards for a positive effect (low sugar content, therefore light chip color) and downwards for a negative effect (high sugar content, therefore dark chip color)

^d^Bold SNP markers were in strong linkage disequilibrium, showed similar effects in the trait, and presented similar distributions in the population

**Fig. 1:**
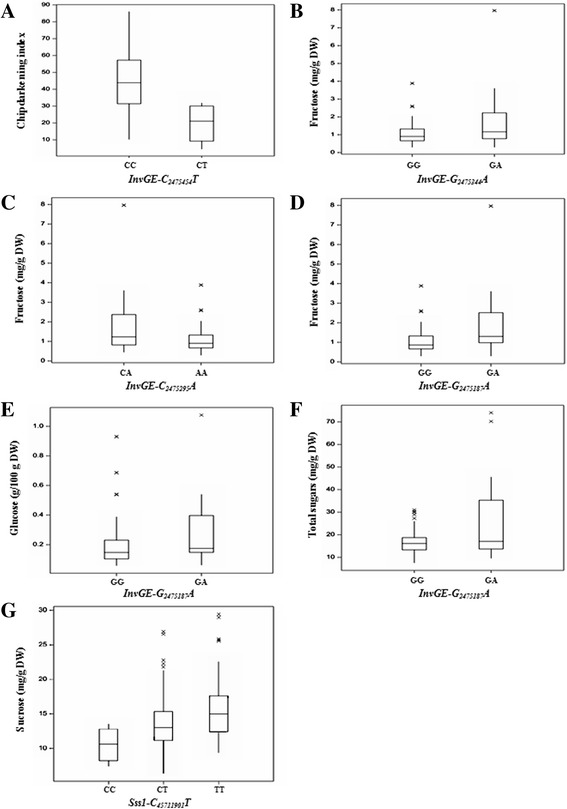
Box plots showing the allele effects of seven significant marker-trait associations: Chip darkening index (**a**), fructose (**b**-**d**), glucose (**e**), total sugars (**f**), and sucrose (**g**). Each plot is labeled with the SNP identification indicated in Table 2. Y-axis: Values for chip darkening index and sugar contents. X-axis: SNP genotypic classes

The box plots in Fig. 1 show the effect of the genotypic classes from markers in the trait variation and supported the significance of the associations. The QQ-plots showed that the models adjusted for each phenotypic trait were effective in controlling type I error (Additional file 1). Model 1 was used for the analysis of chip darkening index and total sugars, while model 2 was used for the analysis of fructose, glucose, and sucrose contents.

Four markers were found in the *InvGE* gene coding for an apoplastic invertase on potato chromosome IX. The average distance between adjacent markers was 88 base pairs (Additional file 2). Three of these markers were previously reported by Draffehn et al. [39] in the analysis of cDNA sequences from exon 3 in tetraploid cultivars and diploid genotypes from an experimental population. *InvGE*-*G*_*2475344*_ *A*, *InvGE*-*C*_*2475295*_ *A*, and *InvGE*-*G*_*2475187*_ *A* corresponded to the markers snp688, snp639, and snp531 from the previous study, respectively. Two of the *InvGE* SNPs are synonymous and the other two are predicted to generate amino acid changes. *InvGE*-*G*_*2475344*_ *A* caused a substitution of non-polar alanine by polar threonine, while *InvGE*-*C*_*2475295*_ *A* causes a substitution of asparagine by lysine, both polar amino acids. The SNP *InvGE*-*C*_*2475454*_ *T* was not found in the analysis of cDNA sequences of this exon [39]. It is therefore a novel variant found in Group Phureja which is associated with chip color.

The minor allele frequency of the SNPs in the *InvGE* gene was not found in the homozygous state in the population (Fig. 1a–f), which indicated that these alleles correspond to low-frequency variants [52]. The SNP *InvGE*-*C*_*2475454*_ *T* presented the minor allele with the lowest frequency and with the greatest effect on the variable of chip darkening index (Table 2, Fig. 1a). The markers *InvGE*-*G*_*2475344*_ *A*, *InvGE*-*C*_*2475295*_ *A*, and *InvGE*-*G*_*2475187*_ *A* (Fig. 1b–f) were in strong LD with an average r^2^ of 0.94 ± 0.02 calculated from the pairwise values. The rare haplotype *InvGE*-*A*_*2475187*_ *C*_*2475295*_ *A*_*2475344*_ showed association with increased fructose content. The SNP *InvGE*-*G*_*2475187*_ *A* from the haplotype block showed multiple significant associations with fructose, glucose and total sugar contents. The corresponding minor frequency allele had a negative effect on the traits (Table 2, Fig. 1d–f). This LD region is supported by the similar effects of the SNPs on the variation of the traits and the similar distributions of the markers in the population (Table 2). Draffehn et al. [39] reported difficulties when sequencing exon 3 in a tetraploid mapping population due to the presence of insertion-deletion polymorphisms. Therefore, the report of this LD region in the exon 3 of *InvGE* and the association of the corresponding rare haplotype with increased sugar contents are novel in cultivated potato.

One SNP marker-trait association was found in an exon of the *SssI* gene coding for a soluble starch synthase on chromosome III (Additional file 2). In this case, the SNP represents a synonymous mutation where all three genotypic classes were observed (Table 2, Fig. 1g). The sequence flanking this marker has not been previously studied in tetraploids [7]. This SNP marker-trait association is therefore novel. The minor allele *SssI*-*C*_*45711901*_ was associated with decreased sucrose content.

## Discussion

The study of ten candidate genes with key functions in carbohydrate metabolism in Group Phureja allowed the identification of novel SNP markers that might be useful for further analyses of these genes in potato. We found SNP markers in two genes that were associated with sugar content and frying color in Group Phureja. These results show additional and novel genetic variability associated with frying quality in potato through the analysis of genes from Group Phureja which were associated in previous studies with sugar content and chip color variation in European tetraploid populations [7, 16, 17, 39]. The association analysis indicates the relevance of the *InvGE* and *SssI* loci in the variation of frying color and sugar content in diverse cultivated potato genetic backgrounds, despite the differences between Group Phureja and European tetraploid germplasm in their physiology and metabolism, ploidy level, allele frequencies and the different strategies for sampling and phenotyping the mapping populations [53].

The study of Group Phureja genotypes with a subtle population structure was appropriate to avoid spurious associations that are caused by highly stratified populations [9, 27]. Despite the current population size might be quite restricted (*n* = 112), the fast LD decay and the high heterozygosity found in the Phureja genotypes supports that the population is highly heterogeneous and provides good resolution for association mapping studies because it is representative of the diversity of this cultivated group [9, 27, 45]. Furthermore, the reliability of the reported associations is supported by the significant effects on the trait variation found with both mixed and linear model approaches.

*InvGE* and *InvGF* in chromosome IX are tandem duplicated genes both coding for apoplastic invertases that form a haplotype block associated with chip quality. They co-localize with the cold-sweetening QTL *Sug9a*, which was highly reproducible among environments [16, 39, 54, 55]. The association of SNP markers in the *InvGE* locus with chip darkening index, fructose, glucose, and total sugar content supports the reproducibility of the association in a diploid potato population. It also supports the correlations among these variables in Group Phureja as discussed previously [36]. These SNPs represent low-frequency variants (Table 2), thus adding evidence that such rare variants can have important effects in complex trait architecture [56].

The current study allowed the analysis of the sequence of exon 3 from *InvGE* in an association mapping population from Group Phureja. The sequence analysis discovered the novel SNP *InvGE*-*C*_*2475454*_ *T* with a significant effect on the variation of the multi-environmental chip darkening index. The HPLC analysis in tubers harvested in an environment representative of Colombian highlands complemented the previous result by allowing the discovery of an *InvGE* haplotype that is associated with a negative effect on fructose content. Since a thorough analysis of this exonic region was not possible in a tetraploid association mapping population [39], it is not possible to establish whether this haplotype is typical for Group Phureja or if it is present also in tetraploid germplasm. The results also show contrasting effects on the trait variation of the marker *InvGE*-*C*_*2475454*_ *T* and the adjacent haplotype, indicating that both regions are inherited independently because they do not form a single LD block. Additional research is required to validate the contrasting effects of the low-frequency variants present in these two gene regions given the outstanding positive effect of the rare allele *InvGE*-*T*_*2475454*_ on light frying color. Consequently, it might be of relevance to generate and select genotypes homozygous for the *T* allele, in order to confirm the effect of this rare allele on light frying color.

The SNP markers *InvGE*-*G*_*2475344*_ *A* and *InvGE*-*C*_*2475295*_ *A* that were predicted to cause amino acid changes are expected to have an impact on protein function. Expression studies in tetraploids detected *InvGE* and *InvGF* transcripts in leaves and flowers but not in mature tubers [57]. In contrast, Maddison et al. [54] found *InvGE* expressed in the tissue surrounding tuber buds. Functional analyses of the corresponding potato alleles were not consistent with their statistical association with chip quality [57]. In fact, apoplastic invertases are relevant in regulating the import of glucose and fructose for tuber initiation and their expression might be time-dependent [58, 59]. Expression analyses have also shown that tissue expression of these invertases is genotype-dependent [57]. Because of the lack of conclusive evidence for a functional association of *InvGE* DNA variants with chip quality, further experiments are required to reveal the potential functional influence of apoplastic invertases on sugar content in mature tubers from Group Phureja. Nevertheless, experimental evidence suggests that the association of this locus is more likely to be indirect as a result of LD with a functional gene [57], which needs to be proven.

The possibility of a haplotype block in the distal region of chromosome IX is supported by the co-segregation of two microsatellite loci in landraces from Argentina. One locus is located in the gene *InvGF* while the other is in the gene *SbeII* that encodes a starch branching enzyme [60, 61]. Although *SbeII* is not predominantly expressed in tubers, antisense inhibition experiments have shown that it has a major effect on starch structure [62, 63]. Recently, an association study linked DNA polymorphisms in this gene to the degree of starch phosphorylation, a process related to starch degradation and sugar accumulation [41]. A linkage disequilibrium region on chromosome IX might contain *InvGE*, *InvGF*, and *SbeII*, the latter potentially having a strong functional association with sugar content in mature tubers. Therefore, it is relevant to explore in Group Phureja the presence of a haplotype block larger than the one reported here for *InvGE*, through the evaluation of markers in *InvGF*, *SbeII*, and other close genomic regions. The identification of associated regions in large haplotype blocks is favorable for breeding purposes because it might allow the design of diagnostic markers with high predictive value due to the low recombination rates in these regions [16].

The association of the SNP *SssI*-*C*_*45711901*_ *T* reflects the effect of a marker with three genotypic classes showing a minor allele with a higher frequency than the minor alleles from markers in *InvGE*. Starch synthases catalyze the glycosyl transfer from ADP-glucose to glucan. The expression of *SssI* in tubers is low, suggesting a minor role of this particular enzyme in starch synthesis in storage organs [64]. As in the previous case, this SNP-trait association might be indirect, resulting from LD with causal variants, or direct through a potential novel role of *SssI* in the control of sugar content in tubers. The SNP in *SssI* is novel since sequence analyses in tetraploids have not reported this nucleotide variation [5]; therefore Group Phureja might be a new source of natural allelic variation in this gene with effect in low sucrose contents. The discovery of variants controlling sucrose content is relevant since the prevention of its accumulation is a strategy that can limit the subsequent accumulation of reducing sugars in tubers [65].

The marker-trait associations found explained a limited amount of the overall trait variability in Group Phureja. Therefore, selecting individuals which combine the superior alleles for these markers will not guarantee per se a better chip quality because there might be other regions influencing the trait, and the interaction with other loci might affect their expression [66]. Nevertheless, increasing the frequency of these alleles with positive effects in potato breeding populations can be a starting point for the marker-assisted improvement of frying quality, as well as for testing the reproducibility of the phenotypic effect of the associated markers in different genetic materials and environments [5]. There might be additional genes from the starch-sugar interconversion pathway associated to frying quality in Group Phureja that reflect the divergent metabolisms of both tetraploid and diploid genotypes. A next step would be the implementation of a genome wide approach to unravel additional genetic mechanisms and novel metabolic pathways underlying the frying quality trait of Group Phureja. These approaches would lead to the application of this knowledge on the genetic architecture of sugar contents and frying color to potato breeding programs.

## Conclusions

The candidate gene approach allowed the identification of novel SNP markers in *InvGE* and *SssI* that were associated with frying quality and sugar contents in Group Phureja. These novel genetic associations need to be validated and afterwards might be useful in potato breeding programs. The results show that some genes related to sugar content and frying color variation are conserved in diploid and tetraploid cultivated potato germplasm. There is a lack of conclusive evidence for a functional association of both genes with frying quality. Therefore, further experiments are required to reveal the potential functional influence of the associated SNPs with sugar contents of mature tubers or to demonstrate that associations are indirect as a result of LD with functional genes. The implementation of a genome wide approach is necessary to analyze comprehensively the genomic regions that control starch-sugar equilibrium. The genome wide study is also required to assess the distribution of LD regions in all the chromosomes to establish the mapping resolution. These strategies will contribute to a broad understanding of the genetic architecture of sugar accumulation and frying color of Group Phureja.

## Additional files

Additional file 1: Quantile-quantile plots of the adjusted mix models for each phenotypic trait. (PDF 303 kb)
Additional file 2: Amplicon sequences with SNP positions in candidate genes with associations. (DOCX 23 kb)
Additional file 3: Amplicon sequences with SNP positions in additional candidate genes studied. (DOCX 26 kb)
