# Correction to: Novel SNP markers in *InvGE* and *SssI* genes are associated with natural variation of sugar contents and frying color in *Solanum tuberosum* Group Phureja

**DOI:** 10.1186/s12863-017-0555-x

**Published:** 2017-10-20

**Authors:** D. Duarte-Delgado, D. Juyó, C. Gebhardt, F. Sarmiento, T. Mosquera-Vásquez

**Affiliations:** 1 Faculty of Agricultural Sciences, Agronomy Department, National University of Colombia, Bogotá, Colombia; 2 Present address: INRES-Plant Breeding, University of Bonn, Bonn, Germany; 3 Department of Plant Breeding and Genetics, Max Planck Institute for Plant Breeding Research, Cologne, Germany; 4 Faculty of Sciences, Biology Department, National University of Colombia, Bogotá, Colombia; 5 Faculty of Agricultural Sciences, Agronomy Department, National University of Colombia, Bogotá, Colombia

## Correction

After the publication of this work [1], we detected some mistakes in the reverse primers of *InvGE* and *BMY-8/2* and in the forward primer of *PWD* shown in Table 1. Additionally, we made some editions in the primer sequences from Additional files 2 and 3. Please see the corrected Table 1 and Additional files 2 and 3 below.

**Table 1:** Loci (PGSC0003DMG*********) from the reference genome [28, 43] analyzed in 112 *Solanum tuberosum* Group Phureja accessions

Gene Acronym	Locus	Chr.	Primer Sequences 5′-3’	Ta (°C)	Amplicon	No. SNPs Scored
(GenBank Accesion No.)					size (bp)	(Scored previously)^e^
*Stp23* (PHO1)^a^ (D00520)	PGSC0003DMG400007782	III	*f-cagatatgttacatactctacc r-tcattagtcacaactttatcgg	59	963	4 (0)
*StpL* (PHO1)^a^ (X73684)	PGSC0003DMG400028382	V	*f-ttacattgcacaagcacaagc r-gtgtacatacaatactctatcc	57	982	14 (1) [67]
*SssI* (Y10416)	PGSC0003DMG402018552	III	f-aacaataggaatttaccataacc *r-atattcccaaacaaaacagagc	57	970	12 (0)
*InvGE* (INV-cw)^a^ (AJ133765)	PGSC0003DMG400008943	IX	f-caattcttcgattcttcatagg *r-acagcacctatgtattataatgg	57	798	9 (8) [39]
*Pain1* (INV)^a^ (X70368)	PGSC0003DMG400013856	III	f-catacattacactatagatcc *r-aattgaagcagatcatgtagg	56	926	5 (2) [39,40]
			f-caaaatgaatacatattaagagg^b^	56	707	4 (0)
			*r-cttaagcagttgcttagagc			
*UGPase* (D00667)	PGSC0003DMG401013333	XI	f-atgatgttctccacttaaaagc	56	805	11 (1)[42]
			*r-tttcagattttcagaagagagg			
			*f-tgattaacgatactatacgtcc	56	931	12 (1) [42]
			r-ttaaaacttccttatactatatgg			
*GWD* (Y09533)	PGSC0003DMG400007677	V	f-ttctgttatctacttagttacg	56	992	5 (3) [7,41]
			*r-gttttatatcttgcttctttgg			
*BMY-8/2* (AF393847)	PGSC0003DMG400001855	VIII	f-gctactggagcatggtgacaga^c^	57	560	9 (9) [5]
			*r-ttacatagaggtctgtcctgcttgag			
*PWD* (AY747068)	PGSC0003DMG400016613	IX	*f-ggtctgatgatctatctgattgc^c^	57	871	16 (16) [7,41]
			r-cgacatcttgaggagaaccaaactt			
*LapN* (X77015)	PGSC0003DMG400007831	XII	f-gcttcctggtcttggctc^d^	60	985	8 (3) [37]
			*r-gataggcatacgcagccaggtcagaaatcaa			

Chr, chromosome; Ta, annealing temperature

* Primer used for amplicon sequencing

^a^ These parenthesis include the alternative acronyms used in the starch-sugar interconversion pathway scheme adapted by Schreiber et al. [7]

^b^ From a region downstream of the *Pain1* candidate gene

^c^ From Schreiber et al. [7]

^d^ From Fischer et al. [37]

^e^ The studies where the SNPs or the related amino acid changes were previously reported in tetraploid potatoes are indicated

## Additional files

Additional file 2: Amplicon sequences of candidate genes with associations to sugar contents and frying color in *Solanum tuberosum* Group Phureja with the SNP alleles and SNP positions in the potato reference genome (version 4.03) [28, 43]. The sequences were retrieved from the SPUD data base [44]. Primer sequences are underlined, markers with associations are highlighted in blue and SNPs previously reported for tetraploid potatoes are shown in italics. The numbers indicate the position of the sequence and the SNPs in the chromosomes (chr). Exonic regions are represented with red letters while introns are represented in black letters according to the gene models from the SPUD data base [44]. (DOCX 23 kb)

Additional file 3: Amplicon sequences of additional candidate genes studied in *Solanum tuberosum* Group Phureja with the SNP alleles and SNP positions in the potato reference genome (version 4.03) [28, 43]. The sequences were retrieved from the SPUD data base [44]. Primer sequences are underlined and the SNPs previously reported for tetraploid potatoes are highlighted in yellow. Exonic regions are represented with red letters while introns are represented in black letters according to the gene models from the SPUD data base [44]. (DOCX 26 kb)
