# Supplementary material for: Novel SNP markers in InvGE and SssI genes are associated with natural variation of sugar contents and frying color in Solanum tuberosum Group Phureja
Source: BMC Genet. 2017 Mar 9;18:23. doi: 10.1186/s12863-017-0489-3 (PMC5345157; doi:10.1186/s12863-017-0489-3)
Supplement: Additional file 1: — Quantile-quantile plots of the adjusted mix models for each phenotypic trait. (PDF 303 kb) [file 12863_2017_489_MOESM1_ESM.pdf]

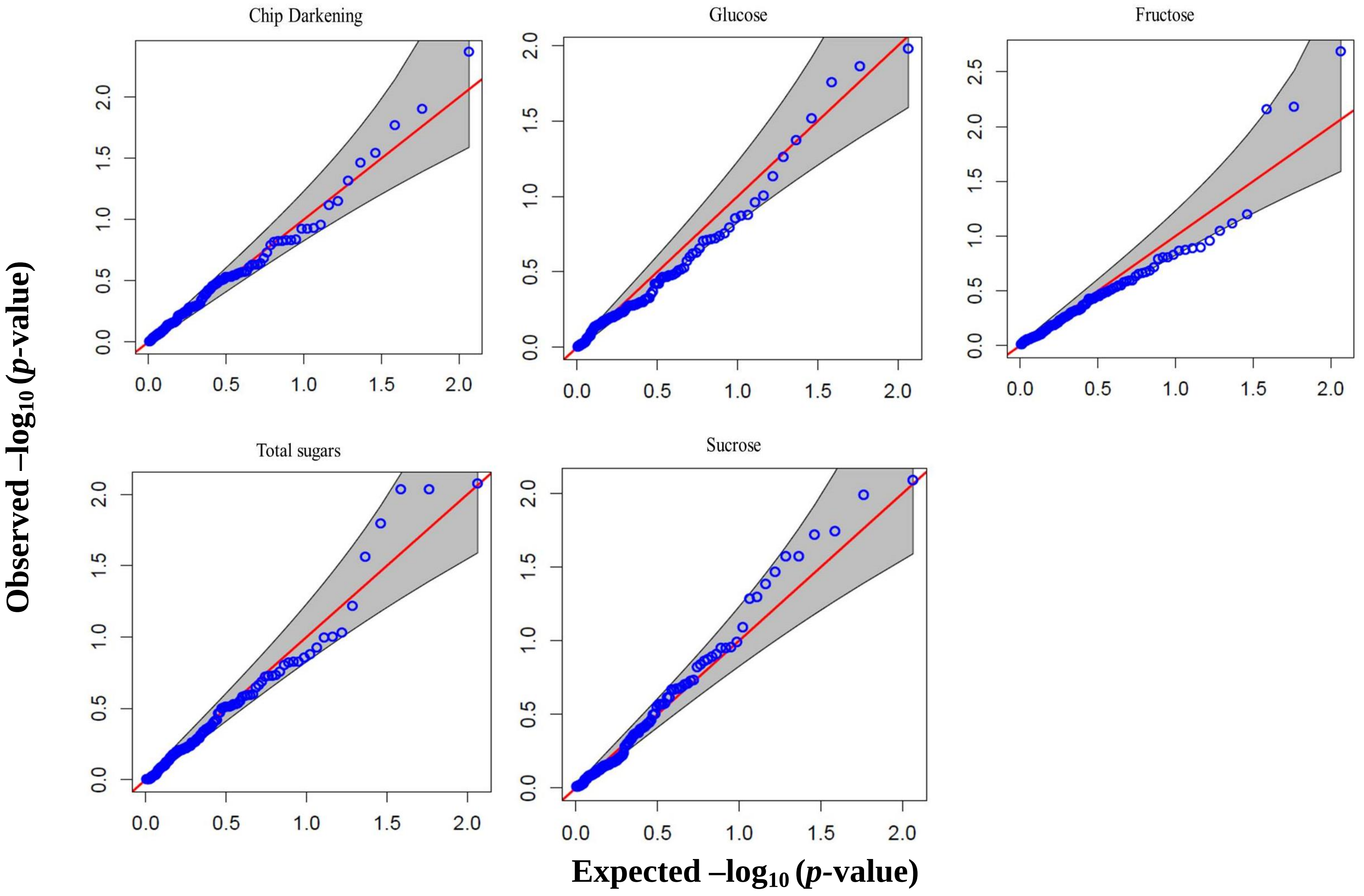

Additional file 1. Quantile-quantile (QQ) plots representing the observed (X-axis) and expected (Y-axis) *F*-test probabilities for the SNP markers tested for association in the traits of chip darkening, glucose, fructose, total sugars, and sucrose contents that showed significant markers in *Solanum tuberosum* Group Phureja. The grey area shows the 95% confidence interval for the QQ-plot under the null hypothesis of no association between the SNP and the trait.
