# Supplementary material for: Correction to: Novel SNP markers in InvGE and SssI genes are associated with natural variation of sugar contents and frying color in Solanum tuberosum Group Phureja
Source: BMC Genet. 2017 Oct 20;18:91. doi: 10.1186/s12863-017-0555-x (PMC5649525; doi:10.1186/s12863-017-0555-x)
Supplement: Supplementary file 1 — Amplicon sequences of candidate genes with associations to sugar contents and frying color in Solanum tuberosum Group Phureja with the SNP alleles and SNP positions in the potato reference genome (version 4.03) [28, 43]. The sequences were retrieved from the SPUD data base [44]. Primer sequences are underlined, markers with associations are highlighted in blue and SNPs previously reported for tetraploid potatoes are shown in italics. The numbers indicate the position of the sequence and the SNPs in the chromosomes (chr). Exonic regions are represented with red letters while introns are represented in black letters according to the gene models from the SPUD data base [44]. (DOCX 23 kb) [file 12863_2017_555_MOESM1_ESM.docx]

**Additional file 2.** Amplicon sequences of candidate genes with associations to sugar contents and frying color in *Solanum tuberosum* Group Phureja with the SNP alleles and SNP positions in the potato reference genome (version 4.03) [28,43]. The sequences were retrieved from the SPUD data base [44]. Primer sequences are underlined, markers with associations are highlighted in blue and SNPs previously reported for tetraploid potatoes are shown in italics. The numbers indicate the position of the sequence and the SNPs in the chromosomes (chr). Exonic regions are represented with red letters while introns are represented in black letters according to the gene models from the SPUD data base [44].

1. ***InvGE*, Apoplastic invertase (PGSC0003DMG400008943)**

chr09: 2474861... 2475657 (reverse complemented)

acagcacctatgtattataatggagtatatcatttattctatcaatacaatccaaaaggatcagtatggggcaatattgtttgggctcattcagtctcaaaagacttgataaattggatccatttagaacccgcaatttatccatctaaaaaatttgacaaatatggtgcttggtccgggtcagcaa

2475067 .5070

ctattctaccaaataacaa[*a/g*]cc[*t/c*]gttatcttatacaccggagtagtagattctcatgattctcaagttcaaaattatgcaatcccggctaact

.5187

tgtctgatccatttcttcgtaaatggatcaaacctaataacaaccc[*g/a*]ttgattgtacctgacaatagcatcaacaaaaccaaatttcgtgatccaaca

.5295

accgcatggatgggccaagatgggctttggagaattgtaataggaagtatgagaaa[*c/a*]catagagggatggcattattgtatagaagtagagact

.5344 .5409

tcattaaatgg[*g/a*]ccaaagcccaacatccacttcattcatctcctcatactggaaattgggaatgtcctgatttttt[*c/t*]cctgtatcattaaaaaata

.5430 .5454 .5470

c[*t/a*]aatggcttagatgcatcgtatcg[c/t]ggaaaaaatgtcaaa[*c/t*]atgtccttaagaatagccttgatgttaataggtttgagtattacactattggtatgtatgacaccaaaaaagataggtacattcctgataacaattctatcgatggttcgaagggattgaggcttgactatgggaatttctatgcatctaagtcattctatgaccctatgaagaatcgaagaattg

1. ***Sss1*, Soluble starch synthase (PGSC0003DMG402018552)**

chr03: 45611303…45612272 (reverse complemented)

45611401

aacaataggaatttaccataaccatatagttgaattctaacatcatcccgaacacctggacagagctcatatagtgtgattaaaaagctaagtgttcc[*t/*

.11441

*c*]agcctctgttcgcttcatcagatacaaacttcagcataa[t/a]gccgtaaacaaagtcatatgatcactttagccatcagaagagcgagtgagcattt

.11549

gacaaactacaaaatacagtgtaactctatccattgagcaaagcttctaata[a/g]ccatccattattcgtagaagttgaggttgttactaaaattggca

.11597 .11608 .11631 .11663

gca[t/c]ggggcattgt[t/a]taatcaaataggaaactatgaa[t/c]tatgatatcttaaaaaacagatttttcacta[a/g]ttagagcaacttgaaaaccatatccggataaaattaaatacaaaaaaattactgaatgttaacttcaaattcaagaaaacaaaaaggggcagtaccagaatgtggaatcaattgaaa

.11838 .11855

accaacaacagagccagaaccatctctctcagcacccaaaagaagcccttctgacac[t/a]ttcttattttcagcaa[t/c]tgatgaaccatctgaaa

.11901 .11937

cactacttgtaacacacaaagattgaac[c/t]tttctgtttcttcgtcccttcaacaaccaagaaaa[t/c]cccaaccccacttgtctttctaccctcaaac

.12051

ccctcacaactctccctgacacacataaacatgacttattgctaagatttgtgggtgtttgcagagaccccatttggtattg[a/g]cacctgctgtaaaatcaagaatcatgtaaagacaaaatctttcaatatagaaaatctgaaaaagtgaaaaaaagtgaaaatgaaacactcattctatattctactactacagtactagtataggatctgatgaagaacacaagtacctagaaagaggtggacagatactcagaacgacatcgttttagtatttgggaaagctctgttttgtttgggaatat
