# Supplementary material for: Correction to: Novel SNP markers in InvGE and SssI genes are associated with natural variation of sugar contents and frying color in Solanum tuberosum Group Phureja
Source: BMC Genet. 2017 Oct 20;18:91. doi: 10.1186/s12863-017-0555-x (PMC5649525; doi:10.1186/s12863-017-0555-x)
Supplement: Supplementary file 2 — Amplicon sequences of additional candidate genes studied in Solanum tuberosum Group Phureja with the SNP alleles and SNP positions in the potato reference genome (version 4.03) [28, 43]. The sequences were retrieved from the SPUD data base [44]. Primer sequences are underlined and the SNPs previously reported for tetraploid potatoes are highlighted in yellow. Exonic regions are represented with red letters while introns are represented in black letters according to the gene models from the SPUD data base [44]. (DOCX 26 kb) [file 12863_2017_555_MOESM2_ESM.docx]

**Additional file 3.** Amplicon sequences of additional candidate genes studied in *Solanum tuberosum* Group Phureja with the SNP alleles and SNP positions in the potato reference genome (version 4.03) [28,43]. The sequences were retrieved from the SPUD data base [44]. Primer sequences are underlined and the SNPs previously reported for tetraploid potatoes are highlighted in yellow. Exonic regions are represented with red letters while introns are represented in black letters according to the gene models from the SPUD data base [44].

1. ***Stp23*, Alpha-1,4 glucan phosphorylase (PGSC0003DMG400007782)**

cagatatgttacatactctaccctggggatgaatcagaggagggaaagatccttcggttgaagcaacaatataccttatgctcggcttctctccaagatattatttctcgatttgagaggagatcaggtgatcgtattaagtgggaagagtttcctgaaaaagttgctgtgcagatgaatgacactcaccctacactttgtatccctgagctgatgagaatattgatagatctgaagggcttgaattggaatgaagcttggaatattactcaaaggtactgaaagttacaagctttgcatgtgggaaattga[a/g]gtttgtgctgaatatactgtac[g/a]gaagcatcatctgaat[c/t]tgtttaaatacatgacgcaagtgatactatagtatgagatcttattactaaactttaagttccacatcatctgaattggtct[c/t]cgaacatcaaaatgacaggagataacacacttttacttttaaacataaaacaatacctccaattttattttcttattagaaacactgaaatccttaatgctggaaacttaccagaactgtggcctacacaaaccatactgttttgcctgaggcactggagaaatggagttatgaattgatgcagaaactgcttcccagacatgtcgaaatcattgaggcgattgacgaggaggtgatggagctacaaatttaacaaatttatttggttatgcaagttaaactattttgactaatttctgcagtgtgctatcctaacagctggtacatgaaattgtattaaaatatggttcaatggatctgaacaaattggaggaaaagttgactacaatgagaatcttagaaaattttgatcttcccagttctgttgctgaattatttattaagcctgaaatctcagttgatgatgatactgaaacagtagaagtccatgacaaagttgaagcttccgataaagttgtgactaatga

1. ***StpL*, Alpha-1,4 glucan phosphorylase (PGSC0003DMG400028382)**

ttacattgcacaagcacaagctcataacaaaactactccctccatttcaatctatatgtctggttttgacttgacacaaag[t/c]ttaagacagtaaaagaaaactcttgaattttgtcatcttatatagttatattaaagatatatagaatgaaagtgcaaaaaaggaaagaggcttcattttggaacg[a/g]actataaagggaagta[a/t][g/a]acaaacaaattgtag[c/t]tgtatcatccttat[g/c][g/a]a[c/t]gaaatgcat[a/c]ttac[c/t]ttatctgttcttca[t/c]gtagtttgtcccggacccaagatttgaagaagtaaagg[t/c]gttcattaggacaggcgtctttggcacctacaactatgaagaactcatgggatccttggaaggaaacgaaggctatggtcgtgctgactattttct[t/a]gtaggaaaggatttccccgattat[a/t]tagagtgccaagataaagttgatgaagcatatcgagaccagaaggtatacttctcattgcatgatctgtctaaccatatctttctctgtttcatattactcgcgcacaacattaggccaagcaaacttattactcatattagtacttagtatccttttcgtgtagcgtttctaaaacatcttatcgttgaaatgaatgcagaaatggaccaaaatgtcgatcttaaacacagctggatcgttcaaatttagcagtgatcgaacaattcatcaatatgcaagagatatatggagaattgaacctgttgaattaccttaaaagttagccagttataggatgaaagccaatttttttcccctgaggttctcccatactgtttattagtacatatattgtcaattgttgctattgaaatgatacaagttttgaatatttactgtcaataaaatacagttgattccatttgaaagtaatgtggtttgttttgaacaacaacatacttgtaattccataggtggagtctggaaaggatagagtattgtatgtacac

1. ***Pain1*, Vacuolar invertase (PGSC0003DMG400013856)**

catacattacactatagatccgccccgctgctgacattgctagacaacaactaaaccttatggcgaatagcaaaagtccagtagaaaaatcactaaaatggcgaataagtagtataacactacatattctccctctcttccctttcttgatgggacatcggtgaaataaccttcaaatgaaaaaaagaatgaagaagatatggcttgatgaattacaagtcttgcaaggggaaggattgaatattagccgactcaagtgaccaaatcttgacggaggcagtcacgctagaccctgtggcattgttgaaaacgaagagtcgtgctgctccattcactgcctttgttgggtaaattcgcgatgttatgactgttcttcctccttgagcaaagctctccacaattgagtggtccacctaaaattcagtttataaaacttttactcaaatgcaactgccaagtactttttttatagtgaaccaactagactacaagaactgaatataagcttgatataaacaaaggtaaataagggaatcatcatcacttaccaataatctcatcgaatgtttttcaccgtccaacacgggtactgaactaccataaacttgtttagcaactcccggagcctctgaggatctgcatatatcagatcataacgcgctacttttatccatctccattttacaaggacta[t/c]ta[t/c]gacaaattaattgtgccagatagagaaaatagaagcaaacctg[g/a]tttgatcagcacagaagtgagtctcagctcg[g/a]ccatcagctcctttagaaatgtagaagtaaactggcgttagctcagatagc[g/t]tttgatcagcaattacaacgacaccaaatggtcccaaaatgcctctgctagcagcacctccactagtagagcagctgaaacctacatgatctgcttcaatt

caaaatgaatacatattaagaggagggaggcgagcgagatcgagagagggaggagagaggattgtattttgtatttattttacattttattttgta[a/t]tattatttagtgcataactgttttgtattttgtatcaattgtattcatcctctctttgattgggtacaattgatacatcatacatttaaaaatgccatatttatacaatgtattttttaccaagttggttcaagtgagcaccctcactccctcctaaattcgaccttgcatatagaattgtatcattgttgtataaaatatgtatccctagagtatgtatatagaagatgaatcattgttgtat[g/a]ttttgtgtaaaataaatatgtaataaatgtataatcagtgttggtcggatgataaacaccattcacctccaaccttaaggttgt[c/t]gctcgggtcacaaaagggaagtaactagggattagtaaaaataactaaataaaaaatatatagaatcaatgtataatgtatacacactaataatataatcttatacagttcttatacactatttatacaca[t/a]gcatgcaagaattatcggaggaaaaatgaagagcgtcaaataagtttatcaaaccagttagttctataccaaacgatcatagatttgatgattttacggtttttatataaactccggcgaaatcacgtgctctaagcaactgcttaag

1. ***UGPase*, UTP—glucose-1-phosphate uridylyltransferase (PGSC0003DMG401013333)**

atgatgttctccacttaaaagcaaagttgtagattgcattttttgtttatcttataagatgcagaaatttcaagttccttttttttttgttgatgcagatcagttattgaagttcgtaatggtttgacattcctcgacttgattgtcaagcaaattgaggtaccacatatcaatggaatattttaatccatcatggtgttccacttttaaatgttctctgattaacttgattaactcgt[a/g]ttctgtaggccctcaatgccaagttcggatgcagtgttcccctgcttttgatgaattcgttcaacacccatgatgatacactgaaggtgaaacaatataactttgatgtcttctcttccatggtt[t/a][t/a]gttttctgtttggctgag[t/c]gc[a/g]agtgctgacttcttaccattgcattctctatgtagattgtagaaaaatatg[t/c]gaactcaaacaattgatattcatacattc[a/g]tcaggtcagtgtgacttatcattagtgatatcaagtatctatcagcttgtatctgca[t/c]gggaaaagttttggatttgtaaagagagatttaa[c/t]gttttctgttaaatattcacagagccagta[c/t]cctcgcctggttacggaagactttgccccacttccatgcaaaggcaattccggaaaagatggatggtaattgacagtttaagatgtgatatttctaatgatgttctttttctttcaaaatgaatcttaattc[g/a]agttctggaatatgtaagctgttgggctctctgttgagttataacgaagtttcatcctctcttctgaaaatctgaaa

tgattaacgatactatacgtcctcagttagcttaaatgatttgtagtgtgctcatccacctgtatat[c/a]tttgcagtctgatctttacaccttgactgatgagggctatgttatccgaaacccg[g/a]ccaggtctaatccatccaacccatccatcgagttgggacctgaattcaagaaggttagcatcaactccgcaagtagattagtgctgcagatttgcatgacatttagctgaatgcagttactgtcttgtctctcaggtggccaacttcttaggccgtttcaagtctattcccagcatcattgatctagatagctt[g/a]aaggtgaccggtgatgtatggttcggatctggcgttaccctcaaggtaattagcaaaactttattctctttctcgagt[a/t]cgtttattttgac[a/g]tgcatttaacgtccta[t/c]caatgacattatccggggaatacaggggaaagtgacc[a/g]ttgc[t/a]gccaaatccggagtgaaactagaaattccagatggtgctgtgattgcaaacaaggtaatacactcgatctgaacgcctaacgcagctac[t/c]ggatttagatttcagtcaggtgtctgatg[t/c]tgtttcattcatttccaggacatcaatggacctgaggatatatagaggagctgttggtgctaattcgacgtcgcggagt[g/c]tgcag[c/t]aagtatgtttgtagtgaatgtaaaatttgtctctttaaataaacagacgttttgcttgttttgatttaatggaaccattgatacactacaaatgaaatgactatatcagagtaacttgttccttcatatttctgtagcttgtctacttcccattttattaatttccatgggaattaatattacaaaaatggaaaagaaattctatatagtaggtcaaaaaatatatataccatatagtataaggaagttttaa

1. ***GWD,* Glucan water dikinase (PGSC0003DMG400007677)**

ttctgttatctacttagttacggatactgtctgttgtgtatttcaggaggaatatgaggctgctcgaactgagctacaggaggaaatagctcgtggtgcttccatacaggacattcgagcaaggctaacaaaaactaatgataaaagtcaaagcaaagaagagcctcttcatgtaacaaagagtgaaatacctgatgaccttgcccaagcacaagcttacattaggtgggagaaagcaggaaagccgaactatcctccagaaaagcaaattgtaaatgctgaacttttcttacagtttatgtagtgtgtgtatatgtctaggcgcataaatctgggtattctgtcttctatagatgtgagactttgatcaagcattgttttattaacaggaagaactcgaagaagcaagaagagaattgcaacttgagcttgagaaaggcattacccttgatgagttgcggaaaaagattacaaaaggggagataaaaactaaggcggaaaagcacgtgaaaagaagctcttttgccgttgaaagaatccaaagaaagaagagagactttgggcagcttattaataagtatccttccagtcctgcagtacaagtacaaaaggtcttggaagaaccacc[a/g]*gccttatctaaaattaagctgtatgccaaggagaaggaggagcagattgatgatccgatcctaaat[g/a]aaaagatctttaaggt[c/a]gatgatggggagctactggtgagtgctaacttattcttgactgtaaccactaatctcagaagctagtttga[a/g]tgcatattgagaatcttccattgtctccacattttaaggtactggtagcaaagtcctctgggaagacaaaagtacat[c/a]tagctacagatctgaatcagccaattactcttcactgggcattatccaaaagtcgtggagagtggatggtaagaataaaaatcgtgcacatcttgcaattgaaaaaaccaaagaagcaagatataaaac

*a/t in Carpenter et al. [41]

1. ***BMY-8/2*, Beta amylase (PGSC0003DMG400001855)**

gctactggagcatggtgacagaatagtagcagcaggagaaagtatataccaaggaactggggctaaactatctggaaaggtagctgggattcattggcattacaatactagatcacatgctgcagagttaacttcaggatattataatacaagacacaga[g/a]atggttatctacctatagcacgtatgttagcgaaacatggtg[c/t]tgtact[t/g]aactttacatgtatggaaatgagggatggtgaacagccccagagtgcaaactgttcaccagaagg[c/a]ttagttc[a/g]acaagttaaaactgcagctagaactgct[a/g]aagtagaacttgctggagaaaatgctctagaaaggtatgatggaggagcattttctcaagtttt[g/a]gcaacaagcatgtca[a/g]attctggaaatggattgagtgcatttacattcttgcgaatgaa[c/t]aaacggttgtttgagccagaaaattggcggaatctagtgcaatttgtgaagagcatgtctgaaggaggtcgaaatgctagccttccagagtgtgactcaagcaggacagacctctatgtaa

1. ***PWD*, Phosphoglucan water dikinase (PGSC0003DMG400016613)**

ggtctgatgatctatctgattgcagtttatagtgaccaaggtgcaccagcatcatttaacgttcctgc[a/t]ggagcagttattccatttggttccatggaaacggcattggaaa[t/c]gaacaagttaatggagaccttcacattgcttgtcgaacagat[a/t]gaaacagctgaaattgatggcggtgaacttgataaaca[t/c]tgtgaggatctccagaagttaatatct[t/a]ctctattgcctggacaagatgtcattgaaagcttgggagaag[t/c]atttcccggtaatgcacgtttaatagtgcgttcaagtgctaatgtcgaggacttggc[g/a]gg[g/a]atgtcagc[t/a]gc[t/c]ggactttatgattcaattcctaatgttagcccttcagatcc[g/a]ataaggtttggacatgctgtagcccgtgtttgggcctcgttgtatactagaagagcagtactgagccgcagagctgctggtgtgtcccagaaaga[c/t]gcta[c/a][a/g]atggc[c/t]gtgctagtgcaagaaatgctttcaccagatttatcttt[c/t]gtcctccacacactgagcccaacggacaacaatcataacttcattgaggctgaaattgcacctggactcggtgaaacactcgcttcaggaacaaggggtacaccgtggcgtctatctagtggtaaatttgacgacacagtgcgcacactggcattcgccaactttagtgaggagatggttgtaggtggcaattcccctgctgacggagaagttattctcttgactgtcgattatagcaagaaacctttaacaatcgaccccattttcagacgtcagcttggtcagaggcttggcgctgttggtttctacctagaacgcaagtttggttctcctcaagatgtcg

1. ***LapN*, Leucine aminopeptidase (PGSC0003DMG400007831)**

gcttcctggtcttggctcaaaaaggattgctctagttgggcttggctcaccaacatcatcaactgctgcttatcgctgtttaggggaggctgctgctgcagctgccaagtctgctcaggctagtaatatcgccattgctcttgcttctacggatggactctctgcagaatcgaagcttagctctgcctctgccataacaactggtatccattttcatggtcttcgcttaattcattgaacc[a/g]tattgagaaactaaacttggttg[a/c]ttttc[g/a]tgaacattgtaggagctgtgctggggacatttgaagataataggtttaa[a/g]tctgagtcaaagaaaccaacattgaaat[c/a]tttggatattcttggactggggactggacctgagatagagaagaaaatcaagtatgcagcagatgtctgtgcaggtgttatactcggaagagagctcgtcaatgcacccgccaatgtacttacgcctggttagtgtttttcaatgcatttccttgttgtcccttttattagtatgcctatc[a/g]ccacactattaaaatgccgacttttcgctgcagcggtacttgctgaagaggccaaaaagattgc[g/a]tccacttatagcgatgtcttttctgcaaacatcttggatgttgagcagtgcaaagaattgaaaatgggatcctatttagc[a/t]gttgctgcagcttctgcaaatcctgctcatttcatccatttgtcttataagcctagtagtggagaaataaaaaagaagatagccttggttggaaagggattaacttttgacaggtaattctatcttctataagttggaaaaatagaaatttgatttctgacctggctgcgtatgcctatc
